# The spectral and spatial distribution of light pollution in the waters of the northern Gulf of Aqaba (Eilat)

**DOI:** 10.1038/srep42329

**Published:** 2017-02-10

**Authors:** Raz Tamir, Amit Lerner, Carynelisa Haspel, Zvy Dubinsky, David Iluz

**Affiliations:** 1 The Mina & Everard Goodman Faculty of Life Sciences, Bar-Ilan University, Ramat-Gan, Israel; 2 The Interuniversity Institute for Marine Sciences of Eilat, Eilat, Israel; 3 Ocean BioOptics and Vision (OBOV) Laboratory, National Institute of Oceanography, Oceanographic and Limnological Research, Tel-Shikmona, P.O.B. 8030, Haifa, Israel; 4 Fredy and Nadine Herrmann Institute of Earth Sciences, The Hebrew University of Jerusalem, Jerusalem, Israel; 5 School of Agriculture and Environmental Studies, Beit Berl College, Kfar Saba, Israel

## Abstract

The urbanization of the shores of the Gulf of Aqaba has exposed the marine environment there, including unique fringing coral reefs, to strong anthropogenic light sources. Here we present the first *in situ* measurements of artificial nighttime light under water in such an ecosystem, with irradiance measured in 12 wavelength bands, at 19 measurement stations spread over 44 square km, and at 30 depths down to 30-m depth. At 1-m depth, we find downwelling irradiance values that vary from 4.6 × 10^−4^ μW cm^−2^ nm^−1^ 500 m from the city to 1 × 10^−6^ μW cm^−2^ nm^−1^ in the center of the gulf (9.5 km from the city) in the yellow channel (589-nm wavelength) and from 1.3 × 10^−4^ μW cm^−2 ^nm^−1^ to 4.3 × 10^−5^ μW cm^−2^ nm^−1^ in the blue channel (443-nm wavelength). Down to 10-m depth, we find downwelling irradiance values that vary from 1 × 10^−6^ μW cm^−2 ^nm^−1^ to 4.6 × 10^−4^ μW cm^−2^ nm^−1^ in the yellow channel and from 2.6 × 10^−5^ μW cm^−2^ nm^−1^ to 1.3 × 10^−4^ μW cm^−2^ nm^−1^ in the blue channel, and we even detected a signal at 30-m depth. This irradiance could influence such biological processes as the tuning of circadian clocks, the synchronization of coral spawning, recruitment and competition, vertical migration of demersal plankton, feeding patterns, and prey/predator visual interactions.

One of the most dramatic changes stemming from the growth in human population and the availability of electricity is the global spread of nighttime illumination^1^. The potential damages of this “light pollution” or “ecological light pollution” phenomenon to ecological systems such as the marine environment are indicated in the very term light pollution^2^,^3^. Due to the increase in human population, coastal habitats adjacent to populated areas have become particularly vulnerable to light pollution^4^,^5^,^6^.

The main sources of anthropogenic light pollution are direct artificial light and sky glow. Sky glow is caused by the scattering of artificial light by the atmosphere and is strongly enhanced by dust, particulate pollution, and reflection by clouds. A prominent component of sky glow is the emission line at a wavelength of 589 nm^7^, mainly from low pressure sodium vapor lighting, which became widespread in the 1960s and 1970s^5^. In addition to sodium vapor lighting, other types of light sources, such as mercury vapor, metal halide, fluorescence, and recently LED based systems, are also in common use (Fig. 1). These sources differ with respect to the spectral distribution of their light emission and consequently with respect to the biological systems they may affect^5^,^7^,^8^. In recent decades, the spectral diversity of artificial light sources has grown, and the trend towards adopting lighting technologies with a broader spectrum of ‘white’ light is likely to increase the potential for ecological impact^5^,^9^. Lighted buildings and towers, streetlights, security lights, boats, flares on off-shore oil platforms, and even lights on undersea research vessels, can all disrupt the ecosystem to varying degrees^2^. The most noticeable effects occur in areas where such lights are close to natural habitats, but even remote areas are exposed to increasing illumination from sky glow, whose impacts are just beginning to be quantified^2^,^9^.

The spread of electric lighting has caused a major perturbation to natural nocturnal light fields and regimes, disrupting the natural cycle of light and darkness, and as a consequence has added a novel environmental stressor, affecting biochemical, physiological, and behavioral patterns that are synchronized with natural diel light/dark field properties. These include, for example, the synchronization of biological clocks^5^,^10^,^11^ and reproductive timing^2^,^12^,^13^,^14^. The ability of artificial light sources to perturb biochemical, physiological, and behavioral patterns is thought to be related to the overlap between the emission spectra of artificial light sources and the absorption spectra of light sensing cryptocromes. (Compare Fig. 1 to http://www.ledgrowlightshq.co.uk/chlorophyll-plant-pigments/). For example, such pigments have been shown to be involved in temporal synchronization processes in corals^10^. It has been suggested that the effects of light pollution on the reproductive physiology, migration, and foraging of marine species could possibly lead to changes in biodiversity^15^ and in the composition of epifaunal communities^8^. Though some ecologists previously acknowledged the potential of artificial nighttime light to disrupt ecological systems in general^2^ and coastal and marine environments in particular^16^,^17^,^18^,^19^, the phenomenon has only recently become widely recognized as an environmental issue^20^, and *in situ* measurements of light pollution and its biological and ecological consequences are lacking.

The natural cycle of light and darkness in the marine environment includes the wax and wane of moonlight, as well as the diel and seasonal patterns of sunlight. The subtle spectral changes in sunlight that take place prior to sunset have recently attracted particular interest as a result of their role in determining the hour of coral spawning^21^. Moonlight is of particular importance, since some marine animals display behavior patterns that are correlated with the synodic monthly phases of the moon^22^. Marine zooplankton have been shown to utilize moonlight for navigation^19^,^23^, for migration^3^, and as a trigger for biological process such as reproduction^11^,^21^,^24^, predator-prey visual interaction^25^, and photosynthesis^26^. Therefore, any perturbation of the natural illumination patterns to which aquatic organisms have adapted in the course of evolution can disrupt their fine-tuned life cycles, behavioral patterns, and physiological mechanisms^2^,^10^,^27^. Anthropogenic light sources constitute a particularly significant perturbation to nighttime underwater illumination, since they operate primarily at night (rather than during the day), and since their light intensity is comparable to that of moonlight (as opposed to that of sunlight).

Particularly vulnerable are light sensitive processes and behavioral patterns of the gulf’s marine organisms, such as the lunar timing of coral spawning, as described by Atoda^28^ and as confirmed in several subsequent studies^29^,^30^,^31^,^32^,^33^, the diel expansion and contraction of tentacles^32^, the diel vertical migration of zooplankton^34^, and the feeding of diurnal corallivorous fishes^35^. Anthropogenic disturbances of the natural light patterns to which organisms have evolved to be attuned to may accelerate the decline of coral reefs that are already stressed by ocean warming, acidification, and eutrophication^36^,^37^,^38^.

The contribution of corals to the primary structural composition of the reef and to the ecology of the reef via their providing an environment for breeding and protection of various organisms, along with the economic value of the corals, has been investigated over the course of a few decades^39^,^40^. However, the extent and potential impacts of light pollution on corals have not been investigated. Functional processes that are affected by environmental light conditions, such as the spawning phase of coral reproduction^10^,^41^, may be disturbed as a result of changes in the levels of light reaching the seabed, which in turn may lead to a lack of synchronization in reproductive processes. In addition, changes in the intensity and spectral quality of artificial light may affect planulae settlement and recruitment patterns of corals in the vertical zonation region of the seabed^16^,^42^, as well as the distribution of coral colony morphology over the area of the reef^43^.

Given that corals are highly photosensitive, with a photoreception sensitivity threshold of blue light of ~1.2 × 10^15^ photons m^−2^ s^−1^ 24, and given previous studies showing that reef structure can be strongly influenced by illumination^44^, there is a definite potential for artificial nighttime lighting to have harmful effects on reef functions and reef health^45^.

Clearly, an understanding of the impact of anthropogenic light sources is crucial for the very survival, sustainable use, management, protection, and bioremediation of important tropical ecosystems, such as coral reefs. As a primary step in this direction, the light field of these sources needs to be mapped in space and time. Previous efforts in this direction have included modeling^46^, remote sensing^6^,^47^, and mapping with geographic information systems (GIS)^48^. However, to the best of our knowledge, the full intensity and spectral composition of nighttime light over the surface of the water and its bathymetric distribution in the water column in a coastal ecosystem have not been previously mapped. The spectral composition of the light is especially important, since different organisms sense light differently, including some organisms that have the ability to sense wavelengths outside of the visible spectrum^2^,^17^.

Here we report for the first time the spatial and spectral distribution of nighttime light in the waters of the Gulf of Aqaba, a complex shoreline containing sectors of industry, commerce, and tourism, and surrounding one of the northernmost flourishing coral reefs on Earth which is protected and managed by the National Parks Authority of Israel.

## The study area

The Gulf of Aqaba (Eilat) is an arm of the Red Sea, surrounded by two cities, Eilat, Israel (34°95′/29°55′), and Aqaba, Jordan (35°00′/29°50′), each with a population of tens of thousands of residents (~60,000 and ~160,000, respectively). In addition, these cities contain industrial complexes along the shores and commercial and military ports, as well as recreational marinas and a thriving tourist industry. As the northern reef destination closest to Europe, the coral reef is the main tourist attraction, with an extremely high number of divers per unit area of reef^49^.

The Gulf of Aqaba is located in a (semi-arid) desert area with strong solar insolation, clear skies throughout most of the year (only ~30 cloudy days yr^−1^, less than 30 mm rain yr^−1^; http://www.iui-eilat.ac.il/Research/NMPMeteoData.aspx), very minimal river runoff, low water turbidity, low re-suspension of sediments^50^,^51^, low nutrient concentration^52^, and low plankton biomass^50^,^52^. These natural conditions allow natural light, as well as different forms of artificial light, to reach and penetrate the water body to considerable depths, with 1% of the subsurface irradiance reaching depths exceeding 110 m^53^. Correspondingly, the vertical attenuation coefficient for underwater downwelling irradiance of photosynthetically active radiation (K_d_ PAR) is low, ranging between 0.04 and 0.065 m^−1^ 50,54,55. As such, the water in the Gulf of Aqaba may be categorized as open ocean, oligotrophic, case I waters^50^,^56^. We also note that the nature of the clear sky in this gulf area may reduce the extent of sky glow^9^, though scattering of artificial light by the atmosphere will still occur to some extent.

Due to the uniquely steep shoreline, the fringing reefs in the gulf skirt the shoreline at the unusually close range of a few meters^57^. Thus, they are particularly exposed to the impact of artificial light sources from the densely populated surrounding urban conglomerations. This is in addition to the combination of tourism, pollution, intensive diving, and shoreline modification, all of which have contributed to the degradation of the gulf’s reefs and the marine ecosystems that are supported by the reefs, as has been observed over the past few decades^57^,^58^.

## Materials and Methods

The underwater downwelling irradiance (*Ed*) in the Gulf of Aqaba was measured on the night of September 9^th^, 2014 at 23:00 local time and on the night of August 12^th^, 2015 at 22:00 local time (GMT + 3.0), using a SeaWiFS-compliant, high resolution, profiling reflectance radiometer (PRR-800; Biospherical Instruments Inc., San Diego) with 19 spectral channels in the 300–900-nm wavelength range. Irradiance in each of the 19 channels, as well as the total irradiance of photosynthetically active radiation (PAR), was measured. The PRR-800 was deployed at nighttime on moonless nights, from a boat, using the free fall technique^59^ in order to avoid shade or reflectance from the boat and in order to keep the light sensor in a vertical posture.

We chose the PRR-800 and its downwelling irradiance mode, because of the ease of deployment, relatively rapid measurement rate for measurements in series at different depths, stability, and compatibility, as well as its highly sensitive sensor. We note that while downwelling irradiance might be the most appropriate variable for estimating the effect of light pollution on corals that live on the sea bed, it is not the most comprehensive measurement for estimating the effect of light pollution on species that detect light incident from all angles (e.g., zooplankton). Nevertheless, since our emphasis in conducting these first measurements of their kind is in evaluating the penetration depth of the light, the downwelling irradiance mode is the most useful for this purpose. Furthermore, since downwelling irradiance is an integral quantity, it is more likely to exhibit stable numbers above the measurement threshold (see more on the measurement threshold in this section) than radiance measurements would.

**Figure 2:**
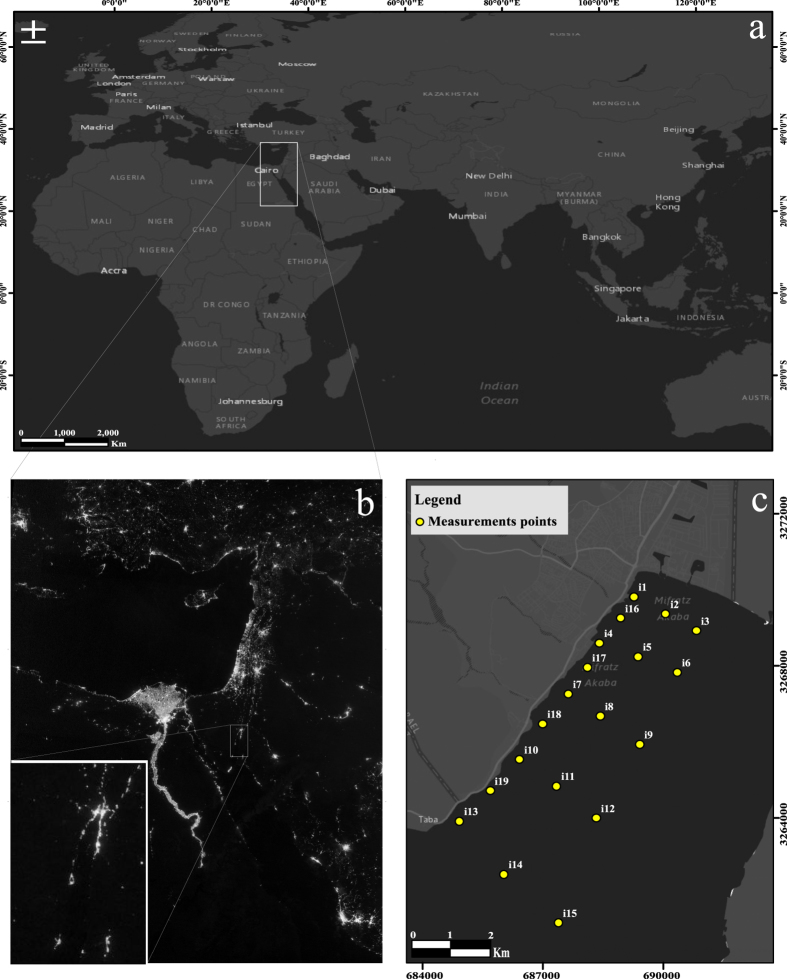
(**a**) World map. (**b**) Night-lights satellite photo of the Middle East and the Gulf of Aqaba close up (Credit: NASA Earth Observatory/Suomi NPP) (**c**) The study area. (**a** and **c** maps were created by using ArcGIS Version. 10.2.1 (Esri Inc.) platform. Sources: Esri, DeLorme, HERE, USGS, Intermap, iPC, NRCAN, Esri Japan, METI, Esri China (Hong Kong), Esri (Thailand), MapmyIndia, TomTom. Esri, DeLorme, HERE, MapmyIndia).

Measurements taken with a value of pitch or roll greater than 10 degrees were removed from the data analysis, in a similar fashion to Wang and Zhao^60^. The instrument was lowered with a velocity of ~0.7 m s^–1^, and irradiance was recorded with a sampling frequency of 5 Hz. Sampling points were distributed throughout the Israeli part of the gulf (Fig. 2). The spatial data were analyzed and presented using the ArcGIS Version. 10.2.1 (Esri Inc.) platform. Spatial interpolation of the data in the horizontal direction was conducted using the standard inverse distance weighting (IDW) method^61^ within the GIS program. We note that the specifications of the PRR-800 are such that noise equivalent irradiance is defined to be 1 × 10^−6^ μW cm^−2^ nm^−1^ or ~1 × 10^−6^ μW cm^−2^ nm^−1^ in a given wavelength channel. This irradiance measurement threshold limited our ability to measure accurate values at greater depths than we present here. Likewise, we found that even at the more highly illuminated stations, for longer wavelength light, we reached the measurement threshold within 1–2 meters below the water surface. Therefore, the red part of the spectrum is not analyzed in the current study despite its potential biological significance. Note that to the best of our knowledge, conducting measurements of light with such a low irradiance remains a technological challenge. We are unaware of any published study in which light in the red part of the spectrum with such low values of irradiance has been successfully recorded.

## Results and Discussion

**Figure 3:**
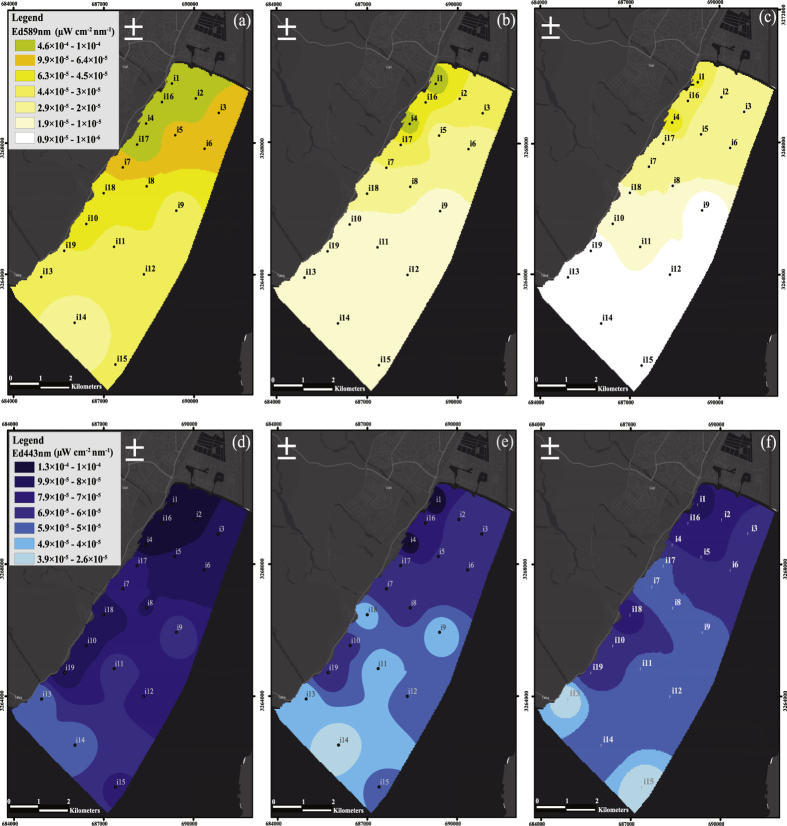
GIS maps of downwelling irradiance, *Ed*, sampled in the Gulf of Aqaba at 1-m, 5-m, and 10-m depth in the wavelength channels of yellow light (589 nm; (**a**–**c)**, respectively) and blue light (443 nm; (**d**–**f**), respectively), on the night of August 12^th^, 2015, at 22:00 local time (GMT + 3). Black/white dots represent sampling locations. The irradiance of yellow light ranged between 1 × 10^−6^ (bright yellow) and 4.6 × 10^−4^ (bright green) μW cm^−2^ nm^−1^. The irradiance of blue light ranged between 2.6 × 10^−5^ (bright blue) and 1.3 × 10^−4^ (dark blue) μW cm^−2^ nm^−1^. (**a**–**f**) maps were created by using ArcGIS Version. 10.2.1 (Esri Inc.) platform. Sources: Esri, DeLorme, HERE, USGS, Intermap, iPC, NRCAN, Esri Japan, METI, Esri China (Hong Kong), Esri (Thailand), MapmyIndia, TomTom. Esri, DeLorme, HERE, MapmyIndia).

**Figure 4:**
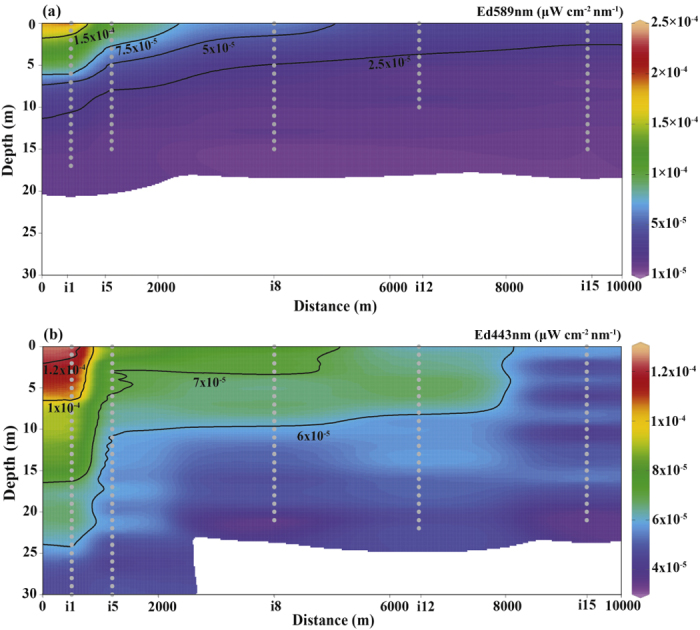
Vertical cross sectional map (depth versus horizontal distance) of downwelling irradiance (*Ed*) in the wavelength channels of (**a**) yellow (589-nm) and (**b**) blue (443-nm) light, sampled on the night of August 12^th^, 2015 at 22:00 local time, in the Gulf of Aqaba water column. The map encompasses measurements at stations i1, i5, i8, i12, and i15 (refer to Fig. 2 for station locations). The x-axis represents the distance from the city of Eilat. The uncertainty in the values of irradiance is ±1 × 10^−6^ μW cm^−2^ nm^−1^. From the surface down to 30 m (the lowest depth at which the instrument was deployed), *Ed* ranged from 2.5 × 10^−4^ to 1 × 10^−6^ μW cm^−2^ nm^−1^ in the 589-nm (yellow) channel and from 1.2 × 10^−4^ to 1 × 10^−6^ μW cm^−2^ nm^−1^ in the 443-nm (blue) channel. Note that the irradiance of the yellow light reached noise level at a depth of 20 m, while the irradiance of blue light reached noise level at a depth of 22–23 m at stations i8, i12, and i15. The white region of the graphs indicates locations at which the measurements were below the measurement threshold. The measurement locations are shown as gray dots.

**Figure 5:**
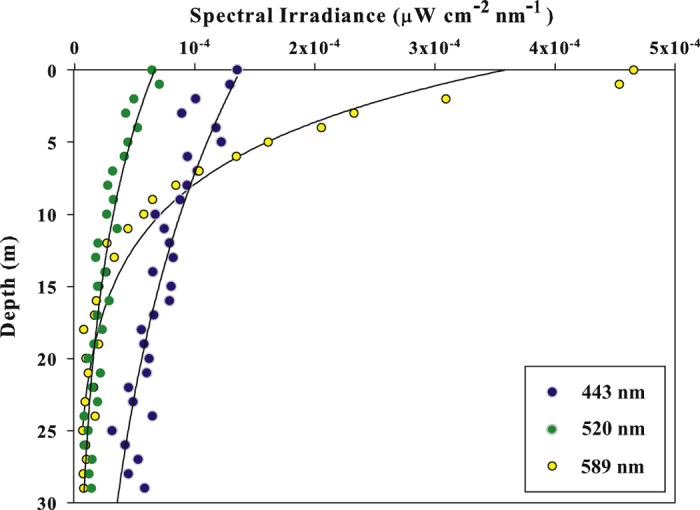
Downwelling irradiance (*Ed*) in the blue (443-nm), green (530-nm), and yellow (589-nm) wavelength channels, respectively, sampled on the night of August 12^th^, 2015, at 22:00 local time, in the Gulf of Aqaba water column at station i4 (see Fig. 2). *Ed* ranged from 4.6 × 10^−4^ to 1 × 10^−6^ μW cm^−2^ nm^−1^ in the 589-nm (yellow) channel, from 7 × 10^−5^ to 1 × 10^−6^ μW cm^−2^ nm^−1^ in the 520-nm (green) channel, and from 1.3 × 10^−4^ to 5.8 × 10^−5^ μW cm^−2^ nm^−1^ in the 443-nm (blue) channel. The irradiance of green and yellow light reached the instrument limit at depths of 15 and 20-m, respectively. The irradiance of blue light did not reach noise level down to the maximum depth to which the instrument was deployed (30 m).

The downwelling irradiance (*Ed*) measurements in the Gulf of Aqaba are shown in Figs 3–5. In Fig. 3, the horizontal variation in downwelling irradiance is shown at three depths in the water column (1 m, 5 m, and 10 m, respectively) and in two wavelength channels [589 nm (yellow light) and 443 nm (blue light), respectively]. In Fig. 4, a vertical cross sectional map (depth versus distance from the shore) of downwelling irradiance in the same two wavelength channels is shown, encompassing measurements from stations i1, i5, i8, i12, and i15 (refer to Fig. 2 for station locations). From Figs 3 and 4, one can see that artificial light from the city of Eilat was detected from the nearest adjacent waters (e.g., station i1 in Fig. 2; 500 m from the city) out to distant points in the center of the gulf (e.g., station i15 in Fig. 2; 9.5 km from the city). In addition to light from the city of Eilat, Eilat’s main port, located less than 300 m from station i4 in Fig. 2, the oil jetty terminal’s offshore pier, which is also located close to station i4, and light sources from the city of Aqaba also contribute to the field of unnatural light in the gulf.

From Fig. 3 and 4, there is a clear horizontal gradient of irradiance, with horizontal differences of more than 1.5 orders of magnitude in the yellow channel and 0.8 orders of magnitude in the blue channel down to 10-m depth. For example, at station i4 at a depth of 1 m under the water’s surface, the measured irradiance in the 589-nm wavelength channel is 4.6 × 10^−4^ μW cm^−2^ nm^−1^ (the maximum value that was measured), while at the most remote station (station i15; 6.3 km from station i1), at the same depth of 1 m, the measured irradiance in the 589-nm wavelength channel is at the measurement threshold of 1 × 10^−6^ μW cm^−2^ nm^−1^. However, due to the multiple light sources contributing to the irradiance at each station, the horizontal gradient is not as steep as would be expected from a one over distance squared dependence from a single localized light source. On the contrary, the irradiance measured at the more remote stations is higher than would be expected from such as simple dependence on distance, thus underscoring the fact that the light pollution is still a significant factor at those relatively large distances. This fact is even more evident from the measured irradiance in the 443-nm wavelength channel, in which the values measured at stations i10 and i19 at a depth of 1 m under the water’s surface (8 × 10^−5^ μW cm^−2^ nm^−1^ and 7.6 × 10^−5^ μW cm^−2^ nm^−1^, respectively) are almost as high as the value measured at station i1 at the same depth (1.3 × 10^−4^ μW cm^−2^ nm^−1^). In addition to the superposition of multiple light sources, sky glow caused by scattering of the light in the atmosphere, as mentioned in the Introduction, can also distribute light from the coastal light sources out to the middle of the gulf.

In Fig. 5, a vertical profile of downwelling irradiance at station i4 in three wavelength channels [520 nm (green light), 589 nm (yellow light), and 443 nm (blue light)] is shown. From Fig. 5, one can see that the intensity of the lights of the city of Eilat and its industrial sections, especially in the yellow and blue parts of the emitted light spectrum, is high enough for the light to penetrate beyond the first few meters of the water column. A weak signal (close to the measurement threshold) was even detected at a depth of 30 m. However, at the more remote measuring locations, the signal to noise ratio dropped within a few centimeters below the water surface. As is evident in Fig. 5, at station i4, down to a depth of ~7 m, the irradiance of yellow light was higher than that of blue light, but below a depth of ~7 m, the irradiance of blue light became higher than that of yellow light. The higher rate of decrease of the irradiance of yellow light with depth is due to the correspondingly higher attenuation coefficient of yellow light in such waters^62^ (case I waters; refer to the Introduction) (e.g., K_d_ at 443 nm = 0.035 m^−1^; K_d_ at 589 nm = 0.14 m^−1^ 56), as well as due to the geometrical configuration of the measurements. (At the air-water interface, yellow light is refracted into a direction farther from vertical than blue light, and therefore the yellow light propagates along a longer path from measurement depth to measurement depth under the water. The fact that the sources of light are localized/pseudo-point sources, and that fact that at each station we measured irradiance through a localized area parallel to the water’s surface, also adds a factor of a decrease with distance squared to the cosine of the local zenith angle, which only accentuates this same effect. For example, from a rough estimation of the attenuation coefficient based on a combination of our daytime and nighttime irradiance measurements (not shown here), the attenuation coefficient of yellow light is ~0.05 m^−1^ higher at depths of ~5–20 m under the water due to geometrical effects than it would be for a plane parallel light source, while the attenuation coefficient of green light is only ~0.03 m^−1^ higher than it would be for a plane parallel light source and only at depths of ~5–10 m under the water, and the attenuation coefficient of blue light is at most 0.01 m^−1^ higher than it would be for a plane parallel light source and only over a range of a few meters depth).

**Figure 6:**
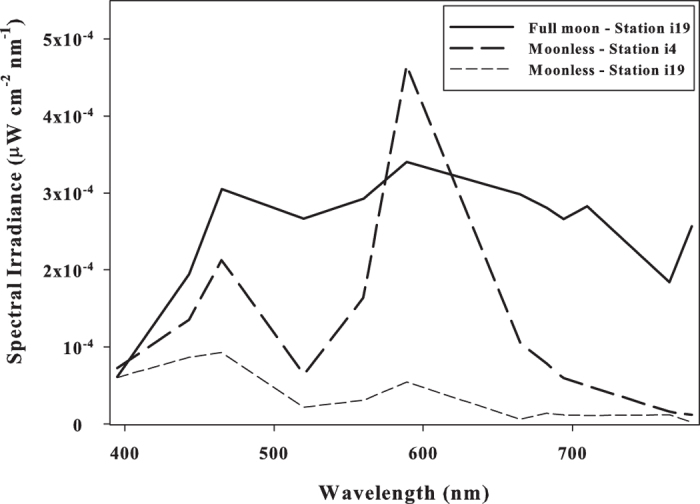
The spectral dependence of measured downwelling irradiance on the water surface on a moonless night (the night of August 12^th^, 2015) at 22:00 local time at a highly illuminated station (station i4; long dashed line) and at a low illuminated station (station i19; short dashed line), and on a full moon night (the night of September 9^th^, 2014) at 23:00 local time at the low illuminated station (station i19; solid line). Stations i4 and i19 are located 150 m and 200 m, from the coastline, respectively.

In Fig. 6, the spectrum of downwelling irradiance on the water surface on the night of August 12^th^, 2015 at 22:00 local time is shown for two locations, a more illuminated station (station i1 in Fig. 2; black curve) and a less illuminated station (station i19 in Fig. 2; gray curve). From Fig. 6, one can see that not only does the absolute value of irradiance differ at the two stations but also the spectral dependence. The peak in the 589-nm wavelength channel due to sodium lighting is especially prominent at station i4, close to the city and the port. In contrast, at the less illuminated station, the blue (443 nm and 465 nm) parts of the spectrum exhibit higher peaks, due to the different types of artificial lighting used in proximity to station i19. Such spectral information at different sites in the gulf is important for assessing the extent and type of impact of the light pollution on the local environment.

Also shown in Fig. 6 are spectral measurements of moonlight on the night of a full moon (the night of September 9^th^, 2014) at 23:00 local time. From Fig. 6, one can see that at station i4 (again near the city and port) on a moonless night, the measured irradiance in the 443-nm and 465-nm wavelength channels are of comparable values to the measured irradiance on the night of the full moon. Furthermore, the irradiance measured in the 589-nm wavelength channel at station i4 on the moonless night is higher than the irradiance measured in the 589-nm channel at station i19 on the night of a full moon. Therefore, artificial nighttime illumination in the Gulf of Aqaba is indeed comparable to and may even exceed the illumination of moonlight. Note that since the intrinsic attenuation coefficient of the water would be the same for both artificial nighttime light and moonlight, and since, as we see from Fig. 6, the irradiance of artificial nighttime light is similar to or larger than the irradiance of moonlight at the surface of the water, the same would be true deeper in the water column where the corals are located.

Our measurements show a clear gradient of unnatural illumination originating from the cities of Eilat and Aqaba and their surroundings into the waters of the Gulf of Aqaba. We have found that in certain wavelength channels, there is almost a two orders of magnitude difference in the irradiance of nighttime light pollution in the Gulf of Aqaba between reef areas next to Eilat and sections of the gulf that are more distant, both at the surface and under water. The fact that the irradiance of artificial light is not uniform over the water body indicates that the effect on water biota may not be uniform. We note that while in terms of vision, a gradient of two orders of magnitude is not extreme (marine animals that navigate vertically through the water column during the day experience similar or larger gradients of light), it is a gradient of light that exists when no light should exist.

The light pollution we have measured in the current study is a potentially harmful factor due to the unique proximity of the coral reefs to the shore and to the urban light sources. The unique geographic setting has to be considered in relation to the manifold different aspects of the influence of light on the structure and functions of coral reefs^33^,^63^,^64^,^65^. This underscores the importance of mapping the spatial and spectral distribution of artificial light in area as Gulf of Aqaba and determining the gradients of light pollution along the gulf. Such mapping provides an experimental framework for the rigorous study of the manifold effects of light pollution on marine life, enabling a comparison of exposed reef sectors to more distant reef sectors as controls. Furthermore, determining the spectral characteristics and sensitivity thresholds of light induced damages and perturbations will provide city planners and municipal lighting designers with critical information necessary to prevent further damage to the gulf’s most important natural treasures and the main socioeconomic resources of the region’s residents.

With respect to the effect of artificial nighttime light on phototroph photosynthesis, Raven and Cockell^26^ measured PAR close to a city and estimated that the combination of full moon light and sky glow (including the contributions of scattering by water vapor and reflection by clouds) could in some cases reach the lower limit required for photosynthesis (0.1 μmol photons m^−2^ s^−1^ 66). At the water surface in the most illuminated locations, we measured a value of total PAR that is slightly above 0.1 μmol photons m^−2^ s^−1^, namely ~0.5 μmol photons m^−2^ s^−1^. Nevertheless, according to our estimation, this total PAR is likely to have a negligible effect on net carbon fixation. [Compare these values to the typical PAR of ~100–2,000 μmol photons m^−2^ s^−1^ that we measured under daylight conditions between the hours of 11:00 and 13:00 local time on a monthly basis from August 2014 to December 2015 using the same methods (results not shown here)]. Under the water surface and at less illuminated locations, where the total PAR measured is even lower, photosynthesis should be completely negligible.

Though the effect of the total PAR on net carbon fixation and photosynthesis is likely to be negligible, the light pollution at the levels reported in the current study may still affect more sensitive physiological and behavioral patterns and processes. These might include synchronization of biological clocks, navigation, migration, reproduction, phototaxis and bioluminescence, and may thereby lead to the impairment of regional marine organisms, as suggested elsewhere (e.g., refs 16 and 67). For example, as we mentioned in the Introduction, several species of corals have been found to be extremely sensitive to the blue region of the light spectrum^10^,^21^,^24^. Moreover, the fact that the irradiance of artificial light at certain stations and in certain wavelengths is similar to or even higher than the irradiance of moonlight on full moon nights (Fig. 6) reinforces the potential disruptive influence of light pollution on the marine ecosystems in the gulf. Therefore, we suggest that future studies of the effect of blue light and of light pollution in general on the specific biological functions of marine organisms and of their ecosystem-level consequences in the Gulf of Aqaba should be given high priority.

## Conclusions

From our *in situ* measurements, we have found that nighttime illumination in the Gulf of Aqaba is dominated by the spectral characteristics of low pressure sodium vapor lights. However, we found that the 589-nm sodium vapor light signature at the surface becomes secondary to the irradiance of blue (443 nm) light from a depth of ~7 m; we found that this 443-nm wavelength light is the most penetrating wavelength in the waters of the gulf. Therefore, the ongoing change in the type of lighting used (the tendency to use more LED lighting, with a stronger blue component) will likely result in a considerable increase in the amount of light that will reach deeper into the water column and to the sea floor, thus exposing more areas of the reef to nighttime light.

We have found that in some locations, the irradiance of near shore artificial light is equal to or exceeds the irradiance of the light of the full moon. Hence, we expect that nighttime light pollution in those locations will interfere significantly with the activities of marine organisms that are synchronized with the phases of the moon. There is a particular danger of upsetting diurnal light/dark vision based behavioral feeding and feeding avoidance patterns of reef dwelling and pelagic organisms, which would destabilize the ecosystem structure and its functions.

Researchers currently face the challenge of disentangling the cumulative effects of all of the facets of human disturbance on coastal ecosystems with which artificial night lighting is often correlated, such as urban development, noise, exotic invading species, animal harvesting, and resource extraction. Even assessing the separate and combined impacts of direct artificial light and sky glow is not trivial.

By providing researchers and decision makers with hitherto unavailable information about the spatial dispersion of disturbed and relatively pristine reef sections of the Gulf of Aqaba, the data presented in the current study should serve as an important contribution towards further research on light pollution and coral reefs and towards future legislation. We expect that this study will prompt follow-up studies on the effects of light pollution on diverse coastal, shallow water ecosystems, helping to predict future shifts in their structure, assemblages, and function. We also expect that our results will promote future efforts to combine an assessment of the influences of light pollution on the specific physiology and behavior of coral reefs and their denizens with an assessment of the bathymetric and geographic boundaries of such effects. Locally, we expect that this study will serve scientists, governmental and local authorities, and interested NGOs in their efforts to ensure the health of the Gulf of Aqaba’s unique coral reefs. The study will have immediate effects on the design of municipal lighting systems and related legislation. We suggest that as coastal cities such as Aqaba and Eilat continue to develop, measurements of light pollution should be included routinely as part of environmental monitoring protocols on both sides of the gulf, and we hope that this study will provide an impetus for collaboration between scientists in Jordan and Israel on this important topic. We also hope that the unique data presented here will raise general public awareness of the issue of coastal light pollution both locally and globally.

## Additional Information

**How to cite this article**: Tamir, R. *et al*. The spectral and spatial distribution of light pollution in the waters of the northern Gulf of Aqaba (Eilat). *Sci. Rep.* **7**, 42329; doi: 10.1038/srep42329 (2017).

**Publisher's note:** Springer Nature remains neutral with regard to jurisdictional claims in published maps and institutional affiliations.

## Figures and Tables

**Figure 1:**
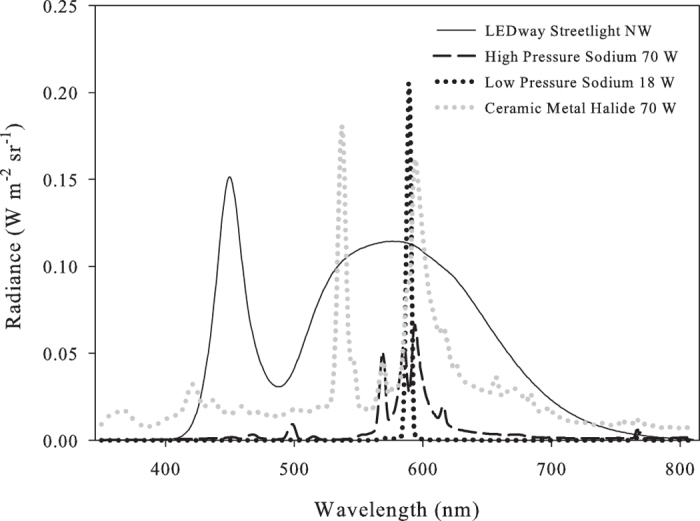
Spectra of different artificial light sources in use: LEDway - white LED (solid black line), high pressure sodium (dashed black line), low pressure sodium (black dotted line), and ceramic metal halide (gray dotted line). The spectra were obtained from http://ngdc.noaa.gov/eog/night_sat/spectra.html.

## References

[b1] Cinzano P., Falchi F. & Elvidge C. D. The first World Atlas of the artificial night sky brightness. Mon. Not. R. Astron. Soc. 328, 689–707 (2001).

[b2] Longcore T. & Rich C. Ecological light pollution. Front. Ecol. Environ. 2, 191–198 (2004).

[b3] Navarro-Barranco C. & Hughes L. E. Effects of light pollution on the emergent fauna of shallow marine ecosystems: Amphipods as a case study. Mar. Pollut. Bull. 94, 235–240 (2015). 25817311 10.1016/j.marpolbul.2015.02.023

[b4] Bird B. L., Branch L. C. & Miller D. L. Effects of coastal lighting on forging behavior of beach mice. Conserv. Biol. 18, 1435–1439 (2004).

[b5] Gaston K. J., Bennie J., Davies T. W. & Hopkins J. The ecological impacts of nighttime light pollution: a mechanistic appraisal. Biol. Rev. 88, 912–927 (2013). 23565807 10.1111/brv.12036

[b6] Falchi F. . The new world atlas of artificial night sky brightness. Science Advances 2, doi: 10.1126/sciadv.1600377 (2016). PMC4928945 27386582

[b7] Morrow J. H. . Advances in measuring the apparent optical properties (AOPs) of optically complex waters. (National Aeronautics and Space Administration, NASA, 2010).

[b8] Davies T. W., Coleman M., Griffith K. M. & Jenkins S. R. Night-time lighting alters the composition of marine epifaunal communities. Biol. Lett. 11, 20150080, doi: 10.1098/rsbl.2015.0080 (2015). 25926694 PMC4424621

[b9] Kyba C. C. M., Ruhtz T., Fischer J. & Holker F. Cloud coverage acts as an amplifier for ecological light pollution in urban ecosystems. PLoS ONE 6, doi: 10.1371/journal.pone.0017307 (2011). PMC3047560 21399694

[b10] Levy O. . Light-responsive cryptochromes from a simple multicellular animal, the coral *Acropora millepora*. Science 318, 467–470, doi: 10.1126/science.1145432 (2007). 17947585

[b11] Takemura A., Ueda S., Hiyakawa N. & Nikaido Y. A direct influence of moonlight intensity on changes in melatonin production by cultured pineal glands of the golden rabbitfish, Siganus guttatus. J. Pineal Res. 40, 236–241 (2006). 16499560 10.1111/j.1600-079X.2005.00306.x

[b12] Rich C. & Longcore T. Ecological Consequences of Artificial Night Lighting. (Island Press, 2006).

[b13] Navara K. J. & Nelson R. J. The dark side of light at night: physiological, epidemiological, and ecological consequences. J. Pineal Res. 43, 215–224 (2007). 17803517 10.1111/j.1600-079X.2007.00473.x

[b14] Holker F., Wolter C., Perkin E. K. & Tockner K. Light pollution as a biodiversity threat. Trends Ecol. Evol. 25, 681–682 (2010). 21035893 10.1016/j.tree.2010.09.007

[b15] Rodrigues P., Aubrecht C., Gil A., Longcore T. & Elvidge C. Remote sensing to map influence of light pollution on Cory’s shearwater in Sao Miguel Island, Azores Archipelago. Eur. J. Wildlife Res. 58, 147–155 (2012).

[b16] Munday P. L., Jones G. P., Ohman M. C. & Kaly U. L. Enhancement of recruitment to coral reefs using light-attractors. Bull. Mar. Sci. 63, 581–588 (1998).

[b17] Gal G., Loew E. R., Rudstam L. G. & Mohammadian A. M. Light and diel vertical migration: spectral sensitivity and light avoidance by *Mysis relicta*. Can. J. Fisheries Aquat. Sci. 56, 311–322 (1999).

[b18] Moore M. V., Pierce S. M., Walsh H. M., Kvalvik S. K. & Lim J. D. Urban light pollution alters the diel vertical migration of *Daphnia*. Verh. Int. Verein. Limnol. 27, 779–782 (2000).

[b19] Kamrowski R. L., Limpus C., Moloney J. & Hamann M. Coastal light pollution and marine turtles: assessing the magnitude of the problem. Endanger Species Res. 19, 85–98 (2012).

[b20] Davies T. W., Duffy J. P., Bennie J. & Gaston K. J. Stemming the tide of light pollution encroaching into marine protected areas. Conserv. Lett. 9, 164–171, doi: 10.1111/conl.12191 (2015).

[b21] Sweeney A. M., Boch C. A., Johnsen S. & Morse D. E. Twilight spectral dynamics and the coral reef invertebrate spawning response. J. Exp. Biol. 214, 770–777, doi: 10.1242/Jeb.043406 (2011). 21307063

[b22] Naylor E. Marine animal behaviour in relation to lunar phase. Earth Moon Planets 85–6, 291–302 (2001).

[b23] Last K. S., Hobbs L., Berge J., Brierley A. S. & Cottier F. Moonlight drives ocean-scale mass vertical migration of zooplankton during the Arctic winter. Curr. Biol. 26, 244–251 (2016). 26774785 10.1016/j.cub.2015.11.038

[b24] Gorbunov M. Y. & Falkowski P. G. Photoreceptors in the cnidarian hosts allow symbiotic corals to sense blue moonlight. Limnol. Oceanogr. 47, 309–315 (2002).

[b25] Gliwicz Z. M. A lunar cycle in zooplankton. Ecology 67, 883–897 (1986).

[b26] Raven J. A. & Cockell C. S. Influence on photosynthesis of starlight, moonlight, planetlight, and light pollution (reflections on photosynthetically active radiation in the universe). Astrobiology 6, 668–675 (2006). 16916290 10.1089/ast.2006.6.668

[b27] Depledge M. H., Godard-Codding C. A. J. & Bowen R. E. Light pollution in the sea. Mar. Pollut. Bull. 60, 1383–1385 (2010). 20736097 10.1016/j.marpolbul.2010.08.002

[b28] Atoda K. The larva and postlarval development of some reef-building corals. I. Pocillopora damicornis Sci. Rep. Tohoku Univ. Fourth Ser. (Biol.) 18, 24–47 (1947).

[b29] Shlesinger Y. & Loya Y. Coral community reproductive patterns: Red Sea versus the Great Barrier Reef. Science 228, 1333–1335 (1985). 17799121 10.1126/science.228.4705.1333

[b30] Babcock R. C. . Synchronous spawning of 105 sclereactinian coral species on the Great Barrier Reef. Mar. Biol. 90, 379–394 (1986).

[b31] Zakai D., Dubinsky Z., Avishai A., Caaras T. & Chadwick N. E. Lunar periodicity of planula release in the reef-building coral *Stylophora pistillata*. Mar. Ecol. Prog. Ser. 311, 93–102 (2006).

[b32] Levy O., Mizrahi L., Chadwick-Furman N. E. & Achituv Y. Factors controlling the expansion behavior of *Favia favus* (Cnidaria: Scleractinia): Effects of light, flow, and planktonic prey. Biol. Bull. 200, 118–126 (2001). 11341573 10.2307/1543305

[b33] Dubinsky Z. & Iluz D. In The Cnidaria, Past, Present and Future: The World of Medusa and her Sisters (eds Goffredo S. & Dubinsky Z.) 469–487 (Springer, 2016).

[b34] Heidelberg K. B., Sebens K. P. & Purcell J. E. Composition and sources of near reef zooplankton on a Jamaican forereef along with implications for coral feeding. Coral Reefs 23, 263–276 (2004).

[b35] Job S. D. & Shand J. Spectral sensitivity of larval and juvenile coral reef fishes: implications for feeding in a variable light environment. Mar. Ecol. Prog. Ser. 214, 267–277 (2001).

[b36] Hoegh-Guldberg O. & Bruno J. F. The impact of climate change on the world’s marine ecosystems. Science 328, 1523–1528 (2010). 20558709 10.1126/science.1189930

[b37] Feely R. A., Doney S. C. & Cooley S. R. Ocean acidification: Present conditions and future changes in a high-CO_2_ world. Oceanography 22, 36–47 (2009).

[b38] Bell P. R. F., Elmetri I. & Lapointe B. E. Evidence of large-scale chronic eutrophication in the Great Barrier Reef: Quantification of chlorophyll a thresholds for sustaining coral reef communities. Ambio 43, 361–376 (2014). 24114070 10.1007/s13280-013-0443-1 PMC3946114

[b39] Moberg F. & Folke C. Ecological goods and services of coral reef ecosystems. Ecol. Econ. 29, 215–233 (1999).

[b40] Lesser M. P. Experimental biology of coral reef ecosystems. J. Exp. Mar. Biol. Ecol. 300, 217–252 (2004).

[b41] Kaniewska P., Alon S., Karako-Lampert S., Hoegh-Guldberg O. & Levy O. Signaling cascades and the importance of moonlight in coral broadcast mass spawning. eLife 4 (2015). 10.7554/eLife.09991 PMC4721961 26668113

[b42] Mundy C. N. & Babcock R. C. Role of light intensity and spectral quality in coral settlement: Implications for depth-dependent settlement? J. Exp. Mar. Biol. Ecol. 223, 235–255 (1998).

[b43] Vermeij M. J. A. & Bak R. P. M. How are coral populations structured by light? Marine light regimes and the distribution of Madracis. Mar. Ecol. Prog. Ser. 233, 105–116 (2002).

[b44] Wellington G. M. An experimental analysis of the effects of light and zooplankton on coral zonation. Oecologia 52, 311–320 (1982). 10.1007/BF00367953 28310389

[b45] Aubrecht C. . A global inventory of coral reef stressors based on satellite observed nighttime lights. Geocarto International 23, 467–479 (2008).

[b46] Chalkias C., Petrakis M., Psiloglou B. & Lianou M. Modelling of light pollution in suburban areas using remotely sensed imagery and GIS. J. Environ. Manage. 79, 57–63 (2006). 16171928 10.1016/j.jenvman.2005.05.015

[b47] Dahdouh-Guebas F. The use of remote sensing and GIS in the sustainable management of tropical coastal ecosystems. Environ. Dev. Sustain. 4, 93–112 (2002).

[b48] Elsahragty M. & Kim J.-L. Assessment and strategies to reduce light pollution using geographic information systems. Procedia Eng. 118, 479–488 (2015).

[b49] Wielgus J., Chadwick-Furman N. E., Dubinsky Z., Schechter M. & Zeitouni N. Dose-response modeling of recreationally important coral-reef attributes: a review and potential application to the economic valuation of damage. Coral Reefs 21, 253–259 (2002).

[b50] Stambler N. Light and picophytoplankton in the Gulf of Eilat (Aqaba). J. Geophys. Res. 111, (C11): Art. No. C11009, doi: 10.1029/2005JC003373 (2006).

[b51] Katz T. . Desert flash floods form hyperpycnal flows in the coral-rich Gulf of Aqaba, Red Sea. Earth Planet. Sc. Lett. 417, 87–98 (2015).

[b52] Lazar B. . In Aqaba-Eilat, the Improbable Gulf. Environment, Biodiversity and Preservation (ed. Por F. D.) 49–62 (Magnes Press, 2008).

[b53] Iluz D. The light field, phytoplankton pigmentation and productivity in the Gulf of Eilat (Hebrew with English abstract), Bar-Ilan University (1997).

[b54] Iluz D., Yehoshua Y. & Dubinsky Z. Quantum yields of phytoplankton photosynthesis in the Gulf of Aqaba (Elat), Northern Red Sea. Isr. J. Plant Sci. 56, 29–36 (2008).

[b55] Dishon G., Dubinsky Z., Fine M. & Iluz D. Underwater light field patterns in subtropical coastal waters: A case study from the Gulf of Eilat (Aqaba). Isr. J. Plant Sci. 60, 265–275 (2012).

[b56] Dishon G. . Optical habitats of ultraphytoplankton groups in the Gulf of Eilat (Aqaba), Northern Red Sea. Int. J. Remote Sens. 33, 2683–2705 (2012).

[b57] Loya Y. The coral reefs of Eilat - past, present and future: Three decades of coral community structure studies. In: Coral Health and Disease (eds Rosenberg E. & Loya Y.) 1–34 (Springer, 2004).

[b58] Loya Y. Changes in a Red Sea coral community structure: A long-term case history study. In The Earth in Transition: Patterns and Processes of Biotic Impoverishment (ed. Woodwell G. M.) 369–383 (Cambridge University Press, 1990).

[b59] Waters K. J., Smith R. C. & Lewis M. R. Avoiding ship-induced light-field perturbation in the determination of oceanic optical properties. Oceanography 3, 18–21 (1990).

[b60] Wang W. B. & Zhao J. P. Variation of diffuse attenuation coefficient of downwelling irradiance in the Arctic Ocean. Acta Oceanol. Sin. 33, 53–62 (2014).

[b61] Bartier P. M. & Keller C. P. Multivariate interpolation to incorporate thematic surface data using inverse distance weighting (IDW). Comput. Geosci. 22, 795–799 (1996).

[b62] Kirk J. T. O. Light and Photosynthesis in Aquatic Ecosystems, 3rd edition. (Cambridge University Press, 2011).

[b63] Falkowski P. G., Jokiel P. L. & Kinzie R. A. Irradiance and corals. In Coral Reefs. Ecosystems of the World (ed. Dubinsky Z.) 89–107 (Elsevier Science Publishers, 1990).

[b64] Dubinsky Z. & Falkowski P. G. In Coral Reefs: An Ecosystem in Transition (eds Dubinsky Z. & Stambler N.) 107–118 (Springer, 2011).

[b65] Iluz D. Coral photobiology: New light on old views. Zoology 118, 71–78 (2015). 25467066 10.1016/j.zool.2014.08.003

[b66] Cockell C. S. & Raven J. A. Zones of photosynthetic potential on Mars and the early Earth. Icarus 169, 300–310 (2004).

[b67] Karl D. M. & Letelier R. M. Marine habitats. In Encyclopedia of Microbiology (ed. Shaechter M.) 258–277 (Elsevier, 2009).

